# Alpha5 Nicotinic Acetylcholine Receptor Contributes to Nicotine-Induced Lung Cancer Development and Progression

**DOI:** 10.3389/fphar.2017.00573

**Published:** 2017-08-23

**Authors:** Hai-Ji Sun, Yan-Fei Jia, Xiao-Li Ma

**Affiliations:** ^1^Key Laboratory of Animal Resistance Biology of Shandong Province, College of Life Sciences, Shandong Normal University Jinan, China; ^2^Central Laboratory, Jinan Central Hospital Affiliated to Shandong University Jinan, China

**Keywords:** alpha5 nicotinic acetylcholine receptor, cell cycle, tumor growth, survival time, nicotine, non-small cell lung cancer

## Abstract

Nicotine and nicotinic acetylcholine receptors (nAChRs) are considered to be involved in lung cancer risk, onset and progression, but their precise physiological roles in these contexts remain unclear. Our previous studies suggested that α5-nAChR mediates nicotine-induced lung cancer cell proliferation, migration, and invasion *in vitro*. In this study, we aimed to determine the role of α5-nAChR in the development and progression of non-small cell lung cancer (NSCLC). Our microarray results reveal that knockdown of the CHRNA5 gene encoding α5-nAChR significantly modulates key pathways including the cell cycle, DNA replication, pathway in cancer. α5-nAChR knockdown in cultured A549 cells affected cell cycle distribution, apoptosis, and cyclin expression. *In vivo*, α5-nAChR silencing inhibited the growth of lung tumors, especially in the context of nicotine exposure. Importantly, α5-nAChR expression in patient tumors correlated with the primary T stage, N stage, and reduced survival time. These results reveal that α5-nAChR silencing inhibits the progression of nicotine-related NSCLC, making this receptor a potential pharmacological target for the treatment of nicotine-related lung carcinogenesis.

## Introduction

Non-small cell lung cancer (NSCLC) accounts for the majority of diagnosed lung cancer subtypes with a relatively poor overall 5-year survival rate (Herbst et al., 2008). Cigarette smoking is the leading risk factor driving lung cancer (Dresler, 2013). Nicotine, is the addictive component of cigarette smoke, and, while not typically thought to be carcinogenic, it has been shown to induce the proliferation, migration, invasion, and survival of cells from multiple cancer types, including lung, pancreas, bladder, head and neck, and gliomas (Grando, 2014; Schaal and Chellappan, 2014). Nicotine exerts these tumor promoting functions through activation of nicotinic acetylcholine receptors (nAChRs) (Carlisle et al., 2007; Schuller, 2009), which can in turn activate either co-receptors or directly enhance downstream signaling cascades.

Nicotinic acetylcholine receptors comprise pentameric subunits, polypeptides that form active receptors using various combinations of 10 α subunits (from α1 to α10), 4 β subunits (from β1 to β4), 1δ, and 1𝜀 or γ subunit, all of which span the plasma membrane. nAChRs are typically expressed at neuromuscular junctions as well as on neuronal cells where they function as ligand-gated ion channels (Itier and Bertrand, 2001; Lee et al., 2011). nAChRs are expressed not only in neuronal systems, but also in numerous non-neuronal tissues tissues, such as lung, pancreas, stomach, head and neck, and gliomas (Calderon et al., 2015; Nieh et al., 2015; Al-Wadei et al., 2016; Jia et al., 2016) indicating that nAChRs likely have roles in other biological processes in addition to synaptic transmission. The full spectrum of biological and physiological functions of individual nAChR subtypes and their individual subunits is far from clear.

Genome-wide association studies (GWAS) have identified a susceptibility locus for human lung cancer at chromosome 15q24-25, which contains the CHRNA3, CHRNA5, and CHRNB4 genes encoding the α3, α5, and β4 subunits of nAChRs (Amos et al., 2008; Hung et al., 2008). Further studies showed that α5-nAChR has been implicated in both nicotine-related behaviors and lung cancer (Jackson et al., 2010; Macqueen et al., 2014). Our own work revealed that the α5-nAChR/HIF-1α/VEGF axis is involved in nicotine-induced tumor cell proliferation and α5-nAChR mediates nicotine-induced cell migration and invasion in NSCLC (Ma et al., 2014; Sun and Ma, 2015). On this basis, further study of α5-nAChRs and their regulation in NSCLC progression is warranted to enable opportunities for the development of new anticancer therapies.

Our microarray profiling coupled with functional pathway analysis revealed that α5-nAChR expression is linked to various cancer-associated pathways in lung cancer cells, especially cell cycle signaling, prompting us to examine the role of α5-nAChR in lung cancer pathogenesis. In this study, we found that lung cancer cells arrest in S phage in response to α5-nAChR downregulation. Moreover, we found that shRNA-mediated down-regulation α5-nAChR in lung cancer cells inhibits tumor formation and α5-nAChR protein expression is significantly associated with NSCLC patient survival.

## Materials and Methods

### Cell Culture and Total RNA Isolation

Human NSCLC cell line A549 was obtained from the American Type Culture Collection and maintained in RPMI 1640 supplemented with 10% fetal bovine serum, penicillin (100 units/mL), and streptomycin (100 Ag/mL; Invitrogen, Corp.) at 37°C in a humidified atmosphere with 5% CO_2_. Previous studies showed that α5-nAChR is expressed in several NSCLC cell lines (Lam et al., 2007). Total RNA was isolated from cells using the RNeasy kit from Qiagen according to the manufacturer’s instructions. Lung adenocarcinoma and normal lung tissue samples were homogenized using Omni plastic disposable probes and an Omni (TH-115) homogenizer (Omni International) for 1 min on dry ice after which total RNA was isolated using Trizol reagent according to the manufacturer’s instructions. Total RNA was quantified using a Nanodrop 1000 spectrophotometer.

### Microarray Processing and Analysis

RNA was isolated from lung cancer cells transfected with scrambled siRNA or α5-nAChR -specific siRNA (three replicates each). Double stranded siRNA oligonucleotides targeting CHRNA5 and a pair of negative control siRNAs were synthesized by GenePharma (China) as previously described (Ma et al., 2014). RNA samples were analyzed by microarray expression profiling using PrimeView Human Gene Expression Array platform (Affymetrix) according to the manufacturer’s instructions (Liu et al., 2014). A total of 2.5 mg of fragmented and labeled cDNA was generated using the Affymetrix GeneChip WT Terminal Labeling and Controls Kit and hybridized onto PrimeView Human Gene Expression Array according to the manufacturer’s instructions (Affymetrix). Arrays were washed, stained, and processed using Affymetrix GeneChip Fluidics Station 450 systems after which they were imaged using Affymetrix GeneChip Scanner 3000 7G for the subsequent generation of raw data. Genes differentially expressed between A549 lung cancer cells transfected with α5-nAChR-specific siRNA compared with cells transfected with scrambled siRNA were selected on the basis of a *P* < 0.001. Gene and functional analysis was conducted using the commercially available software GO & Pathways Analysis according to the manufacturer’s instructions.

### Flow Cytometry Analysis

A549 cells (2 × 10^6^) were plated in 100-mm plates with 15 ml of media, with or without nicotine or α5-nAChR-specific siRNA. Cells were harvested and fixed with 70% cold ethanol at 4°C overnight. After being washed in PBS, the cells were incubated in 1 mL of staining solution (20 mg/mL propidium iodide; 10 U/mL RNaseA) at room temperature for 30 min. Then, samples were measured using a FACSCalibur flow cytometer (Becton Dickinson, Franklin Lakes, NJ, United States), and the percentage of cells in each phase of the cell cycle was obtained using Modfit software.

For the detection of apoptotic cells, A549 cells were trypsinized (0.25% Trypsin, 2.2 mM EDTA) from plates, washed with PBS, and stained with annexin-V-allophycocyanine (APC) according to the manufacturer’s instructions (BD Biosciences Pharmingen). Cells were analyzed with a FACSCalibur flow cytometer (BD Biosciences).

### Western Blot Analysis

Cell monolayers were washed twice with PBS, harvested, and lysed with ice-cold assay buffer (Sigma-Aldrich) after which protein lysates (20 μg) were subjected to SDS-PAGE and Western blotting. The antibodies used for immunoblotting included those raised against cyclin D1, cyclin E2, cyclin D3 (1:500; Cell Signaling Technology), GAPDH (1:1000; Abcam) and horseradish peroxidase-conjugated anti-mouse/rabbit IgG antibody (1:10000; Santa Cruz Biotechnology). After a final wash, signals were detected with use of an enhanced chemiluminescence kit (Amersham Pharmacia, Buckinghamshire, United Kingdom). GAPDH levels were used as an internal standard.

### Tumor Xenograft Assays

All experimental protocols used were evaluated and approved by the Animal Care and Use Committee of Jinan Central Hospital affiliated to Shandong University. Groups of 4- to 6-week-old Balb/c athymic nude mice were purchased from Beijing HFK Bioscience (Beijing, China). To induce ectopic tumor formation, 2 × 10^6^ cells suspended in 100 μl medium were subcutaneously injected in right flank of the mice (*n* = 6). Tumor dimensions were measured every 3 days, and tumor volumes were calculated using the standard formula: length × width^2^/2. Whole body luciferase bioluminescent images were taken using a Xenogen IVIS-100, after the intraperitoneal injection of D-luciferin substrate (Caliper Life Sciences, 160 μl/mice). At the end of the experiment, mice were sacrificed by cervical vertebra dislocation, and tumors weights were measured after dissection. All experiments were approved by the Experimental Animal Ethics Committee of Shandong University (Jinan, P. R. China).

### Tissue Microarrays (TMA)

Tissue microarrays (HLug-Ade150Sur-02) were from Xinchao Biotechnology, Co., Ltd (Shanghai, China). HLug-Ade150Sur-02 contains 75 lung adenocarcinoma and para-carcinoma specimens collected from 2007 to 2012 with survival time data.

### Immunohistochemistry

Immunohistochemical (IHC) analysis was conducted on histologic sections using purified rabbit polyclonal primary antibodies raised against α5-nAChR (1:100). α5-nAChR expression was determined by the percentage of stained tumor cells and staining intensity as previously described (Ma et al., 2014). We examined at least three different high-power (×200) fields of tumor infiltration. The percentage of positive tumor cells was rated as follows: negative expression, no positive staining; weak expression, ≤10% of cells stained positive; and moderate and strong expression, >10% of cells stained positive. Weak expression was rated as negative, and moderate and strong expression was rated as positive for statistical analysis. Expression was analyzed by two independent investigators who used a multi-headed microscope and were blinded to clinical data with consensus.

### Statistical Analysis

Student’s *t*-test or a one-way ANOVA test was used for univariate analyses. A two-way ANOVA test was used to determine the differences in tumor xenograft volume between groups. Pearson’s correlation test was used to consider correlations between α5-nAChR and cyclin D1. Associations of α5-nAChR IHC protein expression with patient outcome (survival time) was estimated using the Kaplan–Meier method and compared among groups by log-rank statistical tests. Statistical analyses were performed using the Statistical Package of Social Science (SPSS) software, version 18.0. All tests were two-sided. A *P*-value < 0.05 was considered statistically significant.

## Results

### Global Gene Expression Analysis of α5-nAChR Knockdown Reveals Modulation of Key Downstream Pathways in Lung Cancer Cells

**FIGURE 1:**
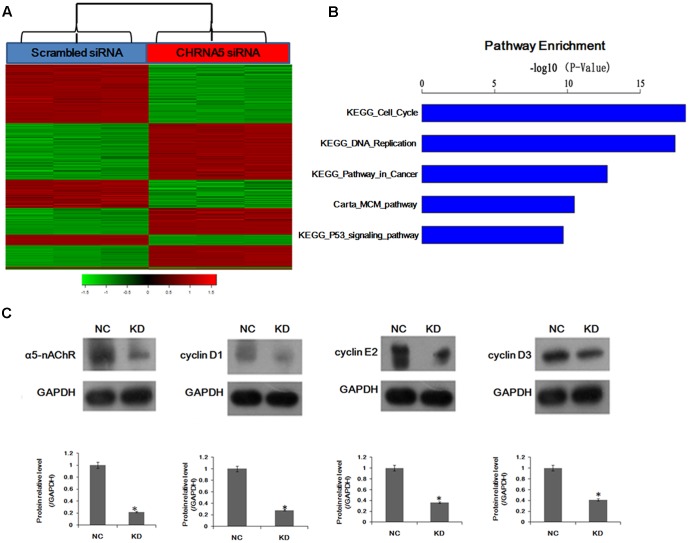
Global changes in the A549 cell transcriptome following knockdown of CHRNA5. **(A)** Heatmap depicting 1,972 transcripts that were significantly differentially expressed between cells transfected with scrambled (Metzger et al., 1976) and CHRNA5-specific (red) siRNA based on *P* < 0.01. Rows and columns represent transcripts and samples, respectively. Upregulated and downregulated gene expression is indicated by red and green colors, respectively. **(B)** Statistically significant modulation of the top five canonical pathways following CHRNA5 knockdown were KEGG_Cell_Cycle, KEGG_DNA_replication, KEGG_Pathway_in_cancer, Carta_MCM_pathway, KEGG_P53_signaling_pathway. **(C)** Confirmation of microarray profiling by Western blotting analysis of cyclin D1, cyclin E2 and cyclin D3 in A549 lung cancer cells transfected with control or CHRNA5-specific siRNA. Expression changes are depicted relative to cells transfected with control siRNA. ^∗^*P* < 0.05.

Our previous studies have showed that α5-nAChR mediates nicotine-induced cell proliferation, migration, and invasion in lung NSCLC *in vitro* (Ma et al., 2014; Sun and Ma, 2015). To gain insights into the mechanisms of α5-nAChR function, we further compared the transcriptome of cells transfected with scrambled siRNA and CHRNA5-specific siRNA. Gene expression profiling, using PrimeView Human Gene Expression Array, identified 1,972 transcripts that were significantly differentially expressed (*P* < 0.01), in CHRNA5 knockdown A549 lung cancer cells compared with control (**Figure 1A**) (Datasets submitted to GEO datasets, with GEO accession: GSE101979). Moreover, pathway analysis of these genes using KEGG^1^ and Biocarta^2^ revealed that CHRNA5 knockdown significantly modulates key pathways, including KEGG_Cell_Cycle, KEGG_DNA_replication, KEGG_Pathway_in_cancer, Carta_MCM _pathway (CDK Regulation of DNA Replication) (Aya-Bonilla et al., 2014), KEGG_P53_signaling_pathway sorting by *P*-value (*P* < 0.01; **Figure 1B**). Notably, pathway analysis revealed that cell cycle signaling was the top modulated canonical pathway following CHRNA5 knockdown (*P* < 0.01; **Figure 1B**). In this regard, we observed significant modulation of the cell cycle genes cyclin D1, cyclin E2 and cyclin D3, which was confirmed by Western blotting (**Figure 1C**). This global transcriptome analysis suggests a functional role for α5-nAChR in human lung cancer cell cycle progression.

### Inhibition of α5-nAChR Regulates Cell Cycle and Apoptosis

**FIGURE 2:**
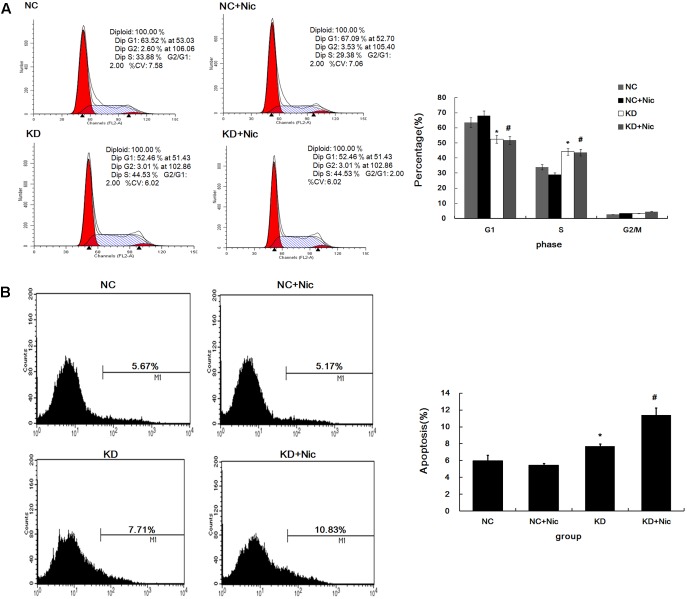
Cell cycle and apoptosis change following α5-nAChR silencing in A549 cells. **(A)** Cell cycle progression of various groups was monitored by fluorescence-activated cell sorting (FACS) analysis (Left) and quantitative analysis of cell cycle distribution is shown (Right). **(B)** A549 apoptosis in various groups was assessed by Annexin V-APC (Left) and quantitative analysis of apoptosis percentage is shown (Right). ^∗^*P* < 0.05 KD vs. NC; #*P* < 0.05 KD+Nic vs. NC+Nic.

To investigate how α5-nAChR affects the NSCLC cell cycle, fluorescence-activated cell sorting (FACS) was used to analyze the cell cycle state of control, nicotine, CHRNA5-siRNA and CHRNA5-siRNA+nicotine groups. As shown in **Figure 2A**, FACS revealed that 52.56 and 51.79% of A549-shCHRNA5 and A549-shCHRNA5+nicotine cells were in G1 phase, and 44.16 and 43.73% were in S phase, respectively. In contrast, 63.50 and 67.99% of the A549-shControl and A549-shControl+nicotine cells, were in G1 phase, while 33.90 and 28.89% were in S phase, respectively (*P* < 0.01). These results suggest that silencing of α5-nAChR expression induces S phase cell cycle arrest.

Furthermore, A549 apoptotic frequencies were 5.67% in the negative control (NC), 5.17% in the NC plus nicotine group (NC+Nic), 7.71% in the CHRNA5 Knockdown group (KD), and 10.83% in the KD plus nicotine group (KD+Nic) (**Figure 2B**). These results indicate that α5-nAChR regulates A549 cell apoptosis.

### Downregulation of α5-nAChR Inhibits Lung Cancer Tumor Formation *In Vivo*

**FIGURE 3:**
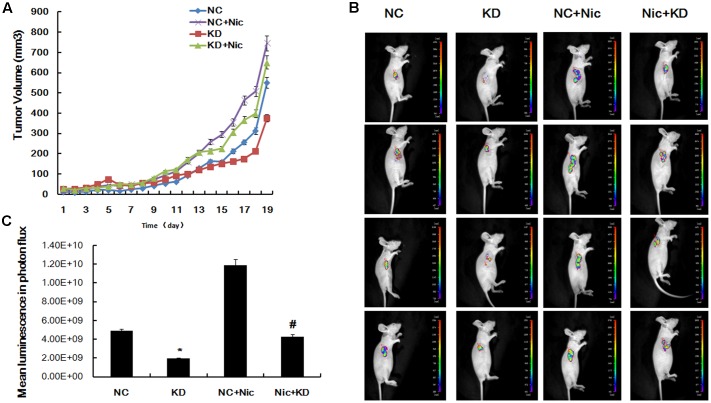
Downregulation of α5-nAChR inhibits lung cancer tumor formation *in vivo.* **(A)** Tumor growth curves were constructed by monitoring tumor size every 2 days post-transplant. **(B)** Tumors xenograft growth was monitored by bioluminescence imaging on day 19 and pictures of four representative mice are shown. **(C)** Mean luminescence of various groups. ^∗^*P* < 0.05 KD vs. NC; #*P* < 0.05 Nic+KD vs. Nic+NC.

Based on our observation of a functional role for CHRNA5 in regulating the cell cycle, proliferation and apoptosis in A549 cells, we next sought to investigate the tumorigenic potential of α5-nAChR expression *in vivo*. A549 cells stably suppressing CHRNA5 or control cells were injected subcutaneously into nude mice. Tumors derived from CHRNA5-suppressing A549 cell clones were measurably smaller than those produced from control cells (**Figure 3A**), and *ex vivo* imaging of organs at the termination of the experiment confirmed these results (**Figure 3B**). Moreover, mice implanted with shcontrol-luc cells that received nicotine had significantly larger tumors compared to those receiving vehicle (**Figure 3C**). Taken together, these results confirm that α5-nAChR is indispensable for optimal lung tumor growth, especially in the context of nicotine exposure.

### α5-nAChR Expression Correlates with Lung Cancer Clinicopathological Variables and Patient Survival

**FIGURE 4:**
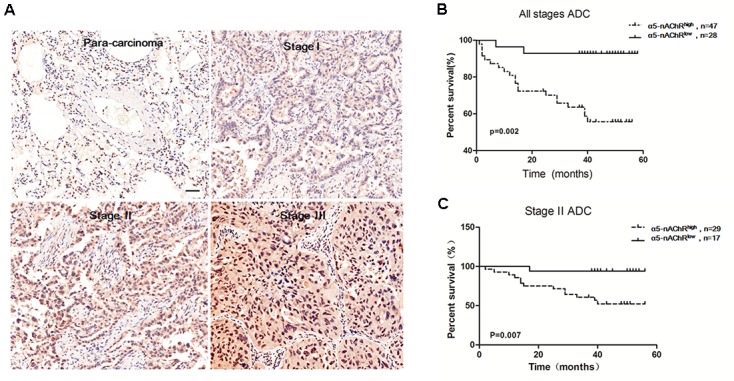
High α5-nAChR expression is associated with shorter survival time in NSCLC patients. **(A)** Overexpression of α5-nAChR in human NSCLC tissues compared to para-carcinoma tissues (200×). Scale bar, 100 μm. **(B)** Survival curve of α5-nAChR high expression compared to α5-nAChR low expression in ADC patients. **(C)** Survival curve of α5-nAChR high expression compared to α5-nAChR low expression for stage II ADC patients.

Our findings demonstrating α5-nAChR’s involvement in lung tumor growth in nude mice prompted us to examine α5-nAChR protein expression as it relates to lung cancer patient outcome. The HLug-Ade150Sur-02 microarray contains 75 specimens of adenocarcinoma and para-carcinoma tissues with survival data (from 2007 to 2012). As measured by immunohistochemistry, α5-nAChR is highly expressed in lung cancer (**Figure 4A**, 62.7%, 47/75) but not in para-carcinoma tissue (**Figure 4A**, 24.0%, 18/75). The association between α5-nAChR expression and clinicopathological variables is shown in **Table 1**. While there is no statistically significant association between α5-nAChR protein expression and age or sex, α5-nAChR expression is correlated with the clinical T and N stages (**Table 1**). Moreover, there is a trend for α5-nAChR expression to correlate with the clinical TMN stage (*P* = 0.083; **Table 1**).

**Table 1:** Associations between α5-nAChR expression and clinicopathological variables.

		α5-nAC		
Clinical	*N*	Positive	Positive rate (%)	*P*
**Age (years)**				
60	37	22	59.46	0.637
>60	38	25	65.79	
**Sex**				
Male	40	27	67.50	0.473
Female	35	20	57.14	
**T stage**				
1	21	11	52.38	0.023
2	39	23	58.97	
3	12	10	83.33	
4	3	3	100.00	
**N stage**				
0	36	21	58.33	0.002
1	7	5	71.43	
2	10	10	100.00	
3	4	4	100.00	
Unknown	18	7	38.89	
**TNM stage**				
I	13	6	46.15	0.083
II	46	29	63.04	
III	16	12	75.00	

GraphPad Prism software was used to analyze the correlation between overall survival and α5-nAChR expression in adenocarcinoma (ADC) patients. This analysis revealed that high α5-nAChR expression patients is predictive of shorter survival time (**Figure 4B**), and, high α5-nAChR expression in Stage II ACD predicts shorter survival time compared with low α5-nAChR expression patients (*P* = 0.007; **Figure 4C**). These results are consistent with data from the Kaplan-Meier plotter web-based tool (KMplot.com) showing that high levels of α5-nAChR correlate with decreased survival probability across all histological subtypes and variants of NSCLC (Schaal and Chellappan, 2016). The association of high α5-nAChR expression with shorter survival time in adenocarcinoma patients suggests a potential tumor-promoting function for α5-nAChR in human lung cancer.

## Discussion

The function of nicotine on cancer cells at the nAChR subtype level is valuable for cancer prevention and treatment (Russo et al., 2011; Wu et al., 2011; Cardinale et al., 2012; Yang et al., 2013). In keratinocytes, α3β2-nAChR and α7-nAChR are necessary for nicotinergic chemokinesis, and α9-nAChR is critical for adhesion and motility (Chernyavsky et al., 2004; Arredondo et al., 2006; Dong et al., 2016). Similarly, in breast epithelial cells α9-nAChR is responsible for nicotine-stimulated cell growth and malignant transformation (Lee et al., 2010; Tu et al., 2011). α5-nAChR was chosen for this study because it has been associated with lung cancer risk and onset, with exposure to a primary cancer etiologic factor (smoking duration and amount), and with the effects of a preventive action (smoking cessation), but its precise function still remains poorly characterized (Jackson et al., 2010; Macqueen et al., 2014; Chen et al., 2016; Hall, 2016). In our previous study, α5-nAChR knockdown abolished nicotine-induced lung cancer cell proliferation and invasion *in vitro*, indicating the importance of the α5-nAChR subtype in lung carcinogenesis. Here, we have further investigated the facilitating effect of α5-nAChR on nicotine-induced NSCLC development and progression.

To gain insights into molecular mechanisms related to α5-nAChR function, we performed gene expression profiling analysis which revealed that CHRNA5 modulated key pathways including cell cycle, DNA replication, pathway in cancer, and α5-nAChR’s effects on cell cycle signaling were confirmed by Western blotting. Moreover, cell cycle analysis was used to demonstrate that α5-nAChR downregulation accelerates the transition of lung cancer cells from the G0/G1 phase to S phase. These results suggest that α5-nAChR might regulate the cell cycle through cell cycle checkpoint proteins. In deed, the expression of cyclin D1, cyclin E2, and cyclin D3 were downregulated in CHRNA5 shRNA transfected lung cancer cells, suggesting that the G0/G1 phase to S phase transition might be regulated by α5-nAChR through cell cyclins. The synthesis of cyclin D is initiated during G1 and drives the G1/S phase transition. Cyclin D1 is a protein involved in the G1/S cell cycle progression, via its participation in complexes with cyclin dependent kinases cdk4, cdk6 and, as a consequence Rb phosphorylation and inhibition of its function (Malumbres et al., 2004). Cyclin D1 is a previously validated nAChR target in normal human bronchial epithelial cells and in some human tumors (Ho et al., 2005; Chen et al., 2010; Chernyavsky et al., 2015). Moreover, nicotine can induce human breast cancer cell proliferation through downregulation of the nicotinic receptor and cyclin D3 (Chen et al., 2011). By far, the underlying molecular mechanisms of nicotine on cell cycle remain unclear (Dasgupta and Chellappan, 2006). To our knowledge, this is the first study to provide evidence that cyclin D1, cyclin E2, and cyclin D3 are targets of α5-nAChR in NSCLC. Further study is needed to assess if α5-nAChR mediates nicotine-induced lung cancer cell proliferation via cyclins (D1/E2/D3) expression.

Published studies have raised the possibility that α5-nAChR might be an important protein in controlling the progression of nicotine-induced lung cancer (Picciotto and Kenny, 2013; Chen et al., 2015; Ray et al., 2017). Indeed, we show here that shRNA-mediated downregulation of α5-nAChR in NSCLC cells inhibits tumor formation *in vivo* and causes increased apoptosis in A549 cells, high α5-nAChR protein expression is predictive of decreased lung cancer patient survival and is correlated with the clinical TMN stage, and, our *in vitro* and *in vivo* experiments support this idea that α5-nAChR is a tumor enhancer for nicotine-induced lung cancer. Several studies have shown that nAChRs regulate the growth and progression of lung (Improgo et al., 2013; Gao et al., 2016) and other cancers (Al-Wadei et al., 2012; Chu et al., 2013; Chernyavsky et al., 2015; Schaal et al., 2015; Zhao et al., 2015; Jia et al., 2016). Our findings here are in accordance with these results and further supported the idea that α5-nAChR mediates nicotine-induced lung cancer development and progression.

## Conclusion

α5-nAChR expression is elevated in lung tumors compared to para-carcinoma tissues and is associated with shorter survival in NSCLC patients. Downregulation of α5-nAChR inhibits lung cancer growth, at least in part, through suppression of cyclins. Together, these findings provide rationale for the further therapeutic targeting of α5-nAChR in nicotine-related NSCLC.

## Author Contributions

H-JS and X-LM: conception and design of the experiments. H-JS, Y-FJ, and X-LM: collection, analysis and interpretation of data. H-JS and X-LM: drafting the article or revising it critically for important intellectual content. All persons designated authors qualify for authorship, and all authors approved the final version of the manuscript for publication.

## Conflict of Interest Statement

The authors declare that the research was conducted in the absence of any commercial or financial relationships that could be construed as a potential conflict of interest.

## Footnotes

1 http://www.genome.jp/kegg/pathway.html

2 https://cgap.nci.nih.gov/Pathways/BioCarta_Pathways
